# Migraine is associated with the development of adult patients with inflammatory bowel disease: a nationwide, population-based study

**DOI:** 10.1038/s41598-024-51455-3

**Published:** 2024-01-12

**Authors:** Chan Hyung Lee, Kyungdo Han, Hyun Jung Lee, Hosun Yu, Seulji Kim, Kookhwan Choi, Seong-Joon Koh, Jong Pil Im, Joo Sung Kim

**Affiliations:** 1 Department of Internal Medicine and Liver Research Institute, Seoul National University College of Medicine, 101 Daehak-ro, Jongno-gu, Seoul, 03080 Korea; 2 Department of Statistics and Actuarial Science, Soongsil University, 369 Sangdo-ro, Dongjak-gu, Seoul, 06978 Republic of Korea

**Keywords:** Inflammatory bowel disease, Migraine

## Abstract

It has been reported that migraine is more common in patients with inflammatory bowel disease (IBD) than in general. However, the impact of migraine on the development of IBD has not yet been elucidated. The aim of this study was to determine the association between migraine and the development of IBD. This nationwide population-based cohort study was conducted using the Korean National Health Insurance Service (NHIS) database. A total of 10,628,070 people aged 20 years or older who had undergone a national health examination conducted by the NHIS in 2009 were followed up until 2017. The study population was divided into two groups according to the presence or absence of migraine. We analyzed the incidence of newly developed IBD, Crohn’s disease (CD), or ulcerative colitis (UC) during the follow-up period. The incidence of IBD was significantly higher in patients with migraine (adjusted hazard ratio [aHR] with 95% confidence interval [95%CI] of 1.31 [1.173–1.468], *p* < 0.001), CD (aHR with 95%CI of 1.58 [1.237–2.013], *p* < 0.001) and UC (aHR with 95%CI of 1.26 [1.106–1.424], *p* < 0.001) than in those without migraine. After 5 years of follow-up, those with migraine showed curves implying cumulative incidences of IBD with a steep increase, especially for CD. In subgroup analysis, migraine was associated with the risk of UC in males (aHR, 1.431 vs. 1.117; interaction *p* = 0.042). Migraine is significantly associated with the development of IBD. Patients with migraine should be monitored carefully for the development of IBD.

## Introduction

Migraine is a recurrent neurological disorder that can seriously affect an individual’s daily life and is still under-recognized and undertreated^1^. Migraine is one of the leading cause of disability around the world in people younger than 50 years old, and 1-year prevalence is estimated at 15% worldwide^2^. Migraine and associated symptoms can be a great burden to patients, impacting function and quality of daily life both during and between migraine attacks^3,4^. This causes tremendous economic losses, where direct and indirect costs were estimated at US$ 11 and 12 billion respectively in 2007^5^. Migraine is frequently accompanied by gastrointestinal symptoms such as nausea, vomiting, dyspepsia, diarrhea, and constipation, and it is also associated with gastrointestinal disorders, including irritable bowel syndrome and inflammatory bowel disease (IBD)^6,7^. This suggests that migraine may be related to interference in the gut–brain axis, which implies that there are reciprocal relationships between neurological and gastrointestinal symptoms^8,9^.

IBD, consisted of Crohn’s disease (CD) and ulcerative colitis (UC), is a chronic and relapsing inflammatory gastrointestinal disorder. In a recent nationwide population-based study, the incidence and prevalence of IBD were shown to be increased in South Korea^10^. Accumulating evidence shows that complex interplay among genetic and environmental factors are associated with the initiation and continuation of gut inflammation^11,12^, and emerging research aims to identify risk factors that could be modified before the onset of IBD. Several studies report an increased risk of migraine among patients with IBD than in the general population, but there is no research about the impact of migraine on the development of IBD^13,14^. Thus, we conducted this nationwide cohort study to determine the association between migraine and the development of IBD.

## Materials and methods

### Data source and study population

This nationwide population-based study was conducted using data from the National Health Insurance Service (NHIS), which is the nationwide obligatory healthcare system for citizens in South Korea^15^. The NHIS database includes demographic data, codes for disease, prescription, and procedure, and records of outpatient and inpatient care. These data are managed with de-identified codes to protect the personal information. The NHIS offers regular national health screenings for early detection of diseases at least once every 2 years. Individuals aged 20 years or older who had received at least one national health screening conducted by the NHIS from January 2009 to December 2009 were enrolled in this study. Patients who were diagnosed with IBD from 2004 until the moment of the health screenings (index date) were excluded. Moreover, individuals diagnosed with IBD in lag period (within 1 year from the index date) or reported with any missing data were excluded. The remaining subjects were followed up from index date until December 2019. Migraine was defined as International Classification of Diseases, 10th revision (ICD-10) code G43 as in previous studies^16–18^, and it was limited to individuals diagnosed at least once in the year of the 2009 health screenings.

### Study endpoint

The primary outcome of this study was newly diagnosed IBD, including CD and UC. The NHIS initiated a registration program for rare intractable disease (RID) as of 2006, where patients with IBD were required to enroll in this program to obtain co-payment reduction. The RID codes were defined by the ICD-10 code and a special code (V code) recorded in the RID database. The reliability of the RID code is high because the subject must be checked for the diagnostic criteria for each RID and be validated by a qualified physician^19^. In the RID program, patients were diagnosed with IBD if they met all strict criteria including clinical manifestation, endoscopic findings, and pathologic findings. Based on the previous studies that proved high accuracy of the incident IBD defined by both ICD-10 and V codes, all patients with IBD were defined by both ICD-10 (CD, K50; UC, K51) and V code (CD, V130; UC, V131) in this study^20,21^. In addition, sensitivity analysis was conducted in Seoul National University Hospital, a tertiary referral hospital in Korea, from January 2010 to March 2013, and the diagnostic sensitivity of CD and UC was determined to be 94.5 and 96.4%, respectively.

### Data collection

Demographic data (age, sex, and residence) and health screening parameters (height, weight, body mass index [BMI], drinking habit, smoking history, regular exercise, and laboratory findings such as fasting blood glucose and glomerular filtration rate [GFR]) of the study population were recorded. Comorbidities, including hypertension (HTN; defined as ICD-10 code I10-13, I15 with antihypertensive drugs or systolic/diastolic blood pressure ≥ 140/90 mmHg), diabetes mellitus (DM; ICD-10 code E11-14 with antihyperglycemic medication or fasting glucose level ≥ 126 mg/dL), dyslipidemia (ICD-10 code E78 with antihyperlipidemic agents or fasting total cholesterol ≥ 240 mg/dL) and chronic kidney disease (CKD; ICD-10 code N18 with kidney damage or GFR < 60 mL/min per 1.73 m^2^ for 3 months or more) were defined using ICD-10 codes, as previously described^22^.

### Statistical analysis

Patient characteristics were compared between the two groups with analysis of continuous variables by t-tests and categorical variables by chi-square tests. Continuous variables were described as mean ± standard deviation (SD) and categorical variables as number and percentage. Cox proportional hazard regression models were preformed to evaluate the risks of newly developed IBD. Results were expressed as hazard ratio (HR) and 95% confidence interval (95%CI). The cumulative incidence for each group was depicted using Kaplan–Meier curves and compared using the log-rank test. The potential effect modification by age, sex, smoking status, drinking habit, regular physical activity, obesity, income, and residence was assessed through stratified analysis and visualized by a forest plot. All statistical analysis were two-tailed, and the statistical significance was defined as p < 0.05. Statistical analyses were performed using the R program version 3.4.3 (The R Foundation for Statistical Computing, Vienna, Austria, http://www.R-project.org) and SAS version 9.2 (SAS Institute Inc., Cary, NC, USA) for Windows.

### Standard protocol approvals, registrations, and patient consents

This study was approved by Seoul National University College of Medicine-Seoul National University Hospital Institutional Review Board (SNUCM-SNUH IRB) on March 24, 2017 (H-1703-107-840) and was conducted in accordance with the Declaration of Helsinki for the participation of human subjects.

## Results

### Baseline characteristics of the study population

A total number of 10,131,193 individuals (male, 55.0%; mean age, 47.08 ± 14.12 years) were enrolled after excluding subjects with missing data (n = 456,374) and patients diagnosed with IBD (n = 40,503) in the designated period. The baseline characteristics according to the presence of migraine are summarized in Table 1. Migraine affected 2.8% of the study population and, compared with controls, migraineurs were more likely to be older and female and to have a rural residence and low income. Moreover, underlying diseases such as metabolic syndrome, HTN, dyslipidemia, and CKD accounted for a higher proportion in patients with migraine.

**Table 1:** Baseline characteristics of study population.

Variables	Migraine	Control	p
	(N = 281,144)	(N = 9,850,049)	
Age (years)	51.7 ± 14.31	46.95 ± 14.09	< 0.0001
< 40	54,596 (19.42)	3,135,896 (31.84)	
< 65	166,734 (59.31)	5,450,739 (55.34)	
65 ≤	59,814 (21.28)	1,263,414 (12.83)	
Sex			< 0.0001
Male	86,745 (30.85)	5,481,768 (55.65)	
Female	194,399 (69.15)	4,368,281 (44.35)	
Place			< 0.0001
Urban	114,974 (40.9)	4,480,892 (45.49)	
Rural	166,170 (59.1)	5,369,157 (54.51)	
Income low (20%)	53,115 (18.89)	1,718,584 (17.45)	< 0.0001
BMI (kg/m^2^)	23.74 ± 3.25	23.71 ± 3.22	< 0.0001
< 18.5	10,459 (3.72)	362,793 (3.68)	
< 23	108,889 (38.73)	3,835,782 (38.94)	
< 25	68,669 (24.42)	2,429,866 (24.67)	
< 30	82,769 (29.44)	2,871,653 (29.15)	
30 ≤	10,358 (3.68)	349,955 (3.55)	
Weight circumference (cm)	79.65 ± 9.13	80.26 ± 9.09	< 0.0001
Drink			< 0.0001
Non-alcoholic	193,609 (68.86)	5,022,782 (50.99)	
Mild-alcoholic	76,033 (27.04)	4,033,731 (40.95)	
Heavy-alcoholic	11,502 (4.09)	793,536 (8.06)	
Smoke			< 0.0001
Non-smoker	215,283 (76.57)	5,803,809 (58.92)	
Ex-smoker	27,390 (9.74)	1,426,674 (14.48)	
Current-smoker	38,471 (13.68)	2,619,566 (26.59)	
Regular exercise	48,241 (17.16)	1,791,388 (18.19)	< 0.0001
Comorbid medical conditions			
Metabolic syndrome	61,317 (21.81)	1,930,185 (19.6)	< 0.0001
Diabetes mellitus	16,514 (5.87)	599,876 (6.09)	< 0.0001
Hypertension	43,665 (15.53)	1,429,690 (14.51)	< 0.0001
Dyslipidemia	36,882 (13.12)	1,093,058 (11.1)	< 0.0001
Chronic kidney disease	24,840 (8.84)	675,317 (6.86)	< 0.0001

IBD, inflammatory bowel disease; CD, Crohn’s disease; UC, ulcerative colitis; GFR, Glomerular filtration rate *Geometric mean (95% CI).

### Incidence and risk of inflammatory bowel disease in patients with migraine

**Figure 1:**
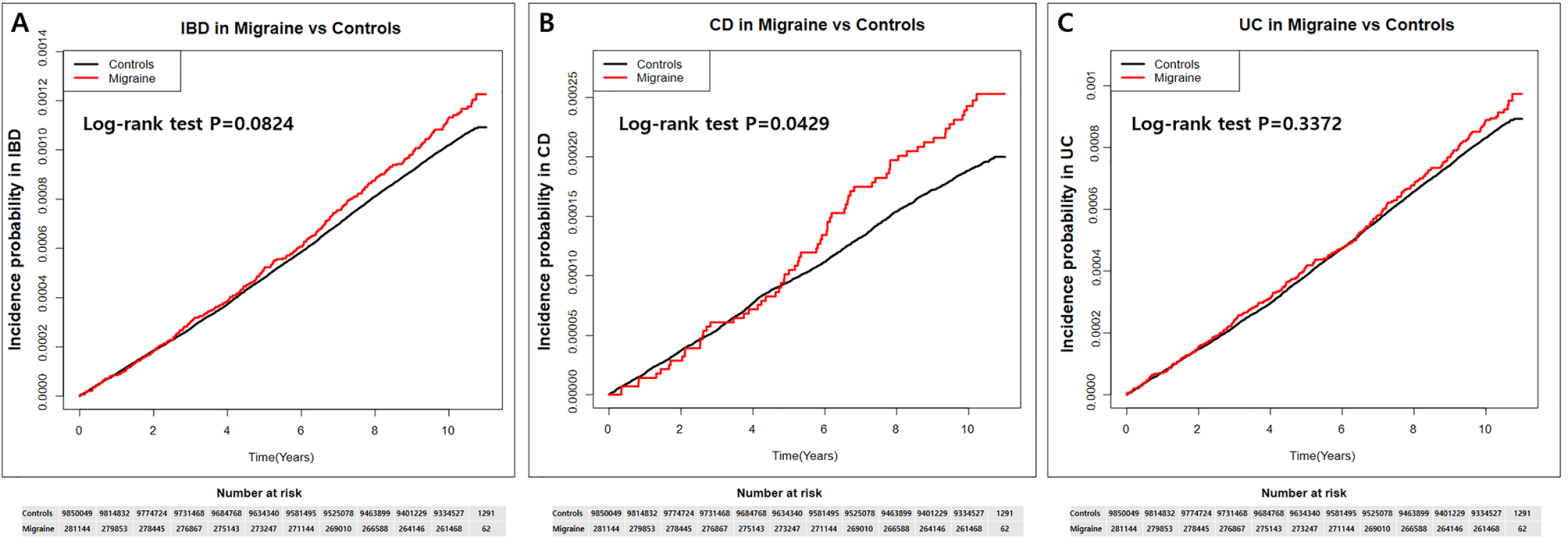
Cumulative incidence of inflammatory bowel disease. Cumulative hazard curves for (**A**) inflammatory bowel disease, (**B**) Crohn’s disease and (**C**) ulcerative colitis in patients with migraine compared to the general population respectively.

During a median follow-up of 10.3 years (interquartile range: 10.11–10.57 years), the incidence rates of IBD per 100,000 person-years were 10.17 and 11.22 in control and migraine groups, respectively (Table 2). The incidence of CD in control and migraine groups was 1.87 and 2.40, respectively, and incidence of UC was 8.30 and 8.82, respectively. Compared with controls, patients with migraine showed a high risk of CD development (crude HR 1.28, 95%CI 1.008–1.635). Moreover, after multivariable adjustment, the risk of IBD and subgroup of CD and UC was also significantly higher in subjects with migraine. Results were adjusted for age, sex, place, income, BMI, smoking, drinking, regular exercise, DM, HTN, dyslipidemia, and CKD. The aHR of IBD increased in patients with migraine (aHR 1.31, 95%CI 1.173–1.468), and similarly, the aHR of CD and UC for patients with migraine showed a significant increase compared to controls (aHR 1.58, 95%CI 1.237–2.013 and aHR 1.26, 95%CI 1.106–1.424, respectively). Cumulative incidence was similar to an increased probability of IBD, CD, and UC in migraineurs, as shown in Fig. 1. Remarkably, the curves implying cumulative incidences showed a steep increase after 5 years of follow-up especially for CD in those with migraine.

**Table 2:** The incidence rate and adjusted hazard ratio of IBD, CD and UC in patients with migraine versus controls.

	Groups	N	Event	Duration	IR per 100,000 p-y	Model1 HR (95% CI)	P	Model2 HR (95% CI)	P	Model3 HR (95% CI)	P-value	Model4 HR (95% CI)	P
IBD													
	Controls	9,850,049	10,142	99,688,759	10.174	1 (Ref.)	0.0825	1 (Ref.)	< 0.0001	1 (Ref.)	< 0.0001	1 (Ref.)	< 0.0001
	Migraine	281,144	318	2,833,947.2	11.221	1.104 (0.987, 1.234)		1.312 (1.172, 1.467)		1.315 (1.175, 1.471)		1.312 (1.173, 1.468)	
CD													
	Controls	9,850,049	1866	99,688,759	1.872	1 (Ref.)	0.043	1 (Ref.)	0.0002	1 (Ref.)	0.0002	1 (Ref.)	0.0002
	Migraine	281,144	68	2,833,947.2	2.399	1.284 (1.008, 1.635)		1.581 (1.239, 2.017)		1.579 (1.238, 2.015)		1.578 (1.237, 2.013)	
UC													
	Controls	9,850,049	8276	99,688,759	8.302	1 (Ref.)	0.3227	1 (Ref.)	0.0005	1 (Ref.)	0.0004	1 (Ref.)	0.0004
	Migraine	281,144	250	2,833,947.2	8.822	1.065 (0.94, 1.208)		1.254 (1.105, 1.423)		1.257 (1.108, 1.427)		1.255 (1.106, 1.424)	

Model1: Non-Adjusted; Model2: Age, Sex adjusted; Model3: Age, Sex, Place, Income, BMI, Smoke, Drink, Regular exercise adjusted; Model4: Age, Sex, Place, Income, BMI, Smoke, Drink, Regular exercise, Diabetes mellitus, Hypertension, Dyslipidemia, Chronic kidney disease adjusted.

CI, confidence interval; HR, hazard ratio; IR, incidence rate; p-y, person-years.

### Subgroup analysis

**Figure 2:**
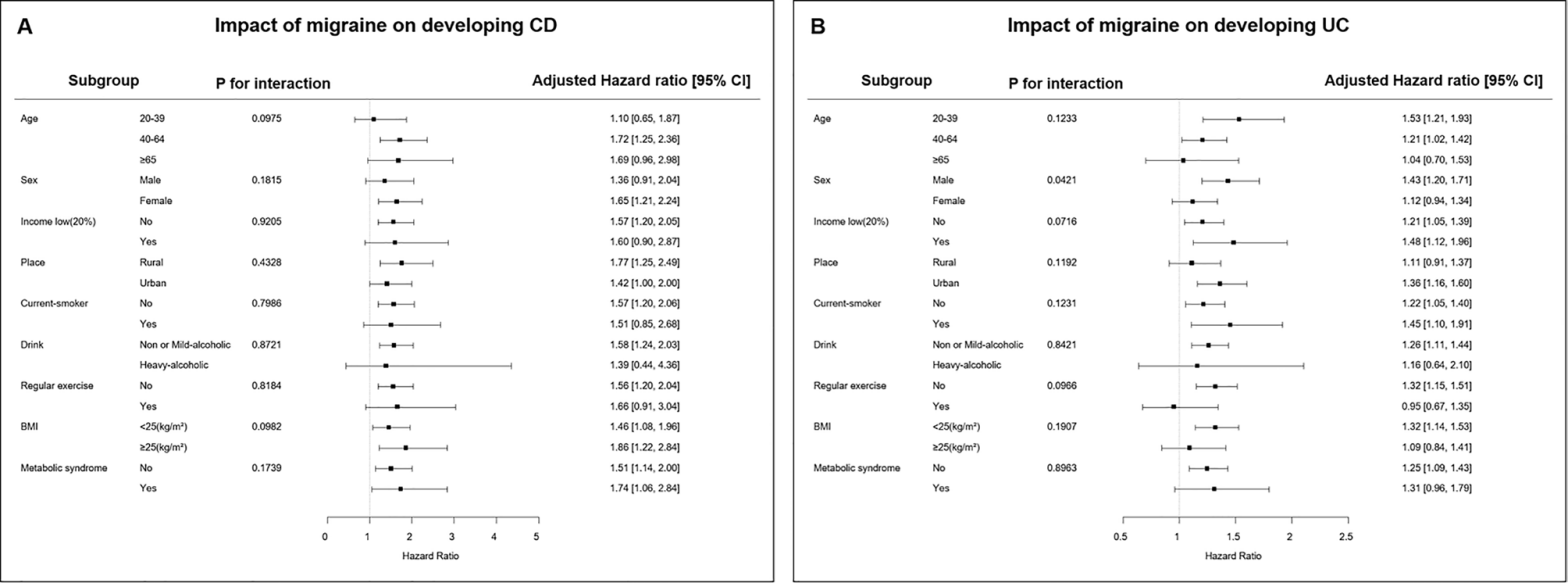
Subgroup analysis for the risk of (**A**) Crohn’s disease and (**B**) ulcerative colitis in patents with migraine compared to control groups.

We revealed the effect of migraine on the incidence of CD and UC according to subgroup assigned by age, sex, income, place, smoking, alcohol, regular exercise, BMI, and presence of metabolic syndrome (Fig. 2). The risk of CD did not show a significant difference between all subgroups. However, the impact of migraine on the risk of UC was more prominent in males than in females (aHR, 1.431 vs. 1.117; interaction *p* = 0.042). The effect of migraine on the development of UC was not significantly different in the other subgroups except sex.

## Discussion

In this nationwide Korean population-based cohort study, we revealed an increased risk for developing IBD including both CD and UC in patients with migraine compared to the general population. To the best of our knowledge, it is the first study to elucidate the impact of migraine on the development of IBD. The cumulative risk of IBD showed a gradual increase following a diagnosis of migraine and, especially for CD, a steep rise after 5 years of follow-up in migraineurs. Moreover, the risk of CD was unaffected by age, sex, health behaviors, or metabolic comorbidities, while the association was prominent in male UC patients. The absolute risk increase of 1/per 100,000 person-years may seem clinically insignificant, but considering inflammatory bowel disease, which has characteristics of very low incidence and prevalence the increase in the hazard ratio of 10% can be regarded as a meaningful increase. Clinically, routine screening could not be recommended for patients with migraine, but prior education and awareness of symptoms associated with IBD may be helpful in the early diagnosis and therapeutic intervention of IBD.

The total prevalence of migraine was 2.8% and the proportion of migraine in females and males was 4.2% and 1.6%, respectively. In line with our results, it is known that migraine is more prevalent in females and that subjects with migraine have higher percentages of rural residency and lower income^23,24^. Furthermore, metabolic syndrome, which involves a number of factors including HTN, hyperlipidemia, obesity, and insulin resistance, is more frequently reported in patients with migraine^25,26^. Previous studies also found an increased risk of metabolic syndrome and cardiovascular disease in patients with IBD^27,28^. However, even after adjusting common risk factors, we demonstrated that the risk for the development of IBD in migraineurs was significantly higher at 1.3-fold compared to the general population.

In addition, in subgroup analysis, the risk of UC in migraineurs was increased by 43% and 12% in males and females, respectively. The significant difference between sexes can be explained by the demographic prevalence of each disease. Higher risk of UC in males can be derived from lower prevalence of migraine and higher prevalence of UC. In comparison between CD and UC among migraineurs, a higher HR was found in CD, which can be supported by several prior studies showing a higher prevalence of CD in patients with migraine compared to UC^29^.

In previous studies, the prevalence of migraine was reported to be higher among those who were formerly diagnosed with IBD. Moreover, IBD was revealed as an independent factor of migraine disorder, suggesting an interaction between gut and brain. Conversely, there have been very few studies investigating the prevalence of IBD in subjects with or without migraine due to the comparatively low prevalence of IBD. In a cross-sectional UK Biobank study, the prevalence of IBD was not significantly higher in the migraine group than in the control group^7^. However, the results of our research re-establish the appropriate temporal relationship as the incidence of IBD is significantly higher in migraineurs using nationwide population-based data. Multifarious mechanisms have been proposed to describe the link between migraine and the development of IBD based on the gut–brain axis. First, proinflammatory cytokines, such as interleukin (IL)-1b, IL-6, IL-8, and tumor necrosis factor-α, have been implicated in migraine pain and are increased during migraine attack, which play a critical role in the pathogenesis of IBD^30^. Second, dysbiosis is known to be involved in the pathophysiology of both migraine and IBD^31^. Psychological and physical stressors could result in changes in the gut microbiota profile, which lead to alterations in intestinal permeability. It is well known that dietary lifestyle, especially the Western diet, is associated with the occurrence of migraine in episodic or chronic forms as well as development of IBD. Still yet to be fully revealed, diet with citrus fruit, processed meat, gluten, chocolate, coffee, and alcoholic beverages are pointed out as dietary risk factors for both migraine and IBD in several studies^32–34^. In addition, migraine attacks could affect the nervous system and play a particular role as a risk factor in the development of IBD. Migraine has long been implicated in impaired gastrointestinal peristalsis, which is induced by sophisticated interactions between the central, enteric, and autonomic nervous system and smooth muscles^35^. Furthermore, several experimental studies identified the impact of decreased gut motility on the microbial environment which is a crucial factor for the development of chronic intestinal inflammation such as IBD^36,37^. Finally, pharmacologic intervention for migraine such as nonsteroidal anti-inflammatory drugs (NSAIDs) has been revealed to have a role in the initiation of IBD. NSAIDs administered at higher frequency for longer duration have been associated with an increased risk of IBD, especially for CD^38,39^, which could be an explanation for the steep rise of cumulative probability after 5 years of follow up.

The first strength of this study was the result of increased risk of IBD in patients with migraine after adjusting for confounding variables, which has the novelty of illuminating the bidirectional gut–brain axis without confinement of cross-sectional analysis. The second strength was the high statistical power that enabled analysis for incidence of IBD in migraineurs. Despite the considerably low incidence and prevalence of IBD, this study of 10,131,193 subjects from NHIS revealed a temporal association between migraine and IBD. Third, the long period of follow-up in this cohort study for obtaining data of incidence was more than 10 years. Moreover, this study was conducted with analysis of data from a qualified public health care system that is capable of controlling the information and selection bias.

This study has several limitations. First, a detailed analysis according to the severity of migraine and IBD could not be evaluated because disease severity data of migraine and IBD was not available from the NHIS database. In addition, the impact of therapeutic modification in patients with migraine on the potential risk of IBD cannot be identified due to restriction of data acquisition. Particularly NSAIDs are well known to be effective in the treatment of migraine attacks, which can affect intestinal permeability and inflammation^40^. Data of NSAIDs including dosage and duration could not be extracted from NHIS database. Further investigations are required to reveal the specific impact on the incidence of IBD based on the difference in severity status of migraine with or without therapeutic intervention. Second, important covariates such as access to healthcare, dietary pattern, scale of stress, family history of IBD and comorbidities of the other gastrointestinal disorders were not included in this analysis. Because of confined adjustment, it is not clear to identify the independent association between prior migraine and risk of IBD. Despite the uncertainty of the pathophysiologic mechanism, the novelty of this study still persists because of the fact that migraine could be regarded as a predictive risk factor implying the higher incidence of IBD in nationwide population-based aspects. Third, a small percentage of the study population was under age of 40 due to complimentary nationwide health screening program provided to individuals of age 40 or above. Although the peak age for IBD is 20 to 40 years, the number of middle‐aged or elderly IBD increased continuously in newly industrialized countries including Korea^41,42^. The proportion of the age would be inappropriate to represent the general population, but may benefit the middle‐aged, socially active population at risk for IBD. In addition, previous study revealed that approximately 28% of patients with CD and 65% of patients with UC were diagnosed after age of 40 in Korea^43^. Finally, because of the retrospective nature, the causal relationship between migraine and IBD could not be determined.

In conclusion, the risk of IBD including CD and UC in patients with migraine was significantly higher compared to the nationwide general population. Therefore, clinicians should be aware of the potential risk of IBD in patients diagnosed with migraine especially in men for the development of UC and in migraineurs with a long disease duration for a further risk of CD. Our findings may pave the way for gut–brain axis to explore the direction and causality of these associations.

## Data Availability

De-identified data from this study will be made available on request after approval by the study investigators. More information about data availability: guswjd80@snu.ac.kr.

## Abbreviations

CD: Crohn’s disease
CI: Confidence interval
CKD: Chronic kidney disease
DM: Diabetes mellitus
HR: Hazard ratio
HTN: Hypertension
IBD: Inflammatory bowel disease
ICD: International classification of disease
NHIS: National healthcare insurance service
RID: Rare intractable disease
SD: Standard deviation
UC: Ulcerative colitis
